# Intersections and Non-Intersections: A Protocol for Identifying Pedestrian Crash Risk Locations in GIS

**DOI:** 10.3390/ijerph16193565

**Published:** 2019-09-24

**Authors:** Mingyu Kang, Anne Vernez Moudon, Haena Kim, Linda Ng Boyle

**Affiliations:** 1 Korea Research Institute for Human Settlements (KRIHS), Sejong-si 30147, Korea; 2 Urban Form Lab and Department of Urban Design and Planning, University of Washington, Seattle, WA 98195, USA; moudon@uw.edu; 3 Department of Civil Engineering, University of Washington, Seattle, WA 98195, USA; haenakim@uw.edu; 4 Department of Industrial & Systems Engineering, University of Washington, Seattle, WA 98195, USA; linda@uw.edu

**Keywords:** pedestrian safety, spatial autocorrelation, algorithm

## Abstract

Intersection and non-intersection locations are commonly used as spatial units of analysis for modeling pedestrian crashes. While both location types have been previously studied, comparing results is difficult given the different data and methods used to identify crash-risk locations. In this study, a systematic and replicable protocol was developed in GIS (Geographic Information System) to create a consistent spatial unit of analysis for use in pedestrian crash modelling. Four publicly accessible datasets were used to identify unique intersection and non-intersection locations: Roadway intersection points, roadway lanes, legal speed limits, and pedestrian crash records. Two algorithms were developed and tested using five search radii (ranging from 20 to 100 m) to assess the protocol reliability. The algorithms, which were designed to identify crash-risk locations at intersection and non-intersection areas detected 87.2% of the pedestrian crash locations (r: 20 m). Agreement rates between algorithm results and the crash data were 94.1% for intersection and 98.0% for non-intersection locations, respectively. The buffer size of 20 m generally showed the highest performance in the analyses. The present protocol offered an efficient and reliable method to create spatial analysis units for pedestrian crash modeling. It provided researchers a cost-effective method to identify unique intersection and non-intersection locations. Additional search radii should be tested in future studies to refine the capture of crash-risk locations.

## 1. Introduction

Promotion of active transportation is an important goal of transportation planning and public health [1,2]. Since walking trips are more likely to be observed in dense urban areas where motorized travel is congested [3,4], a safe environment from motorized vehicles is crucial to protecting pedestrians and promoting walking. Thus, identifying locations where pedestrians are most vulnerable is important to further promote this environmentally friendly and healthy mode of travel. Given the prevalence of motor-vehicles, unprotected pedestrians are vulnerable and prone to experience serious injuries when colliding with motor-vehicles. In 2017, nearly 6000 pedestrians were killed in motor vehicle crashes in the US [5]. While non-pedestrian fatalities decreased by 14% from 2007 to 2016, pedestrian fatalities increased at an alarming rate of 27%.

Studies have shown that the likelihood of pedestrian crashes is impacted by the characteristics of the micro-environment around crash-risk locations (e.g., roadway characteristics and traffic conditions) as well as the characteristics of the macro-environments (e.g., neighborhood characteristics such as development densities and land uses that generate or attract pedestrian travel) [6,7,8,9,10,11]. More specifically, past studies have focused on modelling two types of outcomes: The severity of pedestrian injury and the frequency of pedestrian crashes. While the unit of analysis for injury severity models has been an individual pedestrian crash [7,12,13,14], crash frequency models have adopted location-based approaches. In frequency models, crash locations have been measured as points (e.g., intersection) [6,9], polylines (e.g., roadway segment) [15,16], polygons (e.g., jurisdictional boundary) [17,18], and grid cells (e.g., rasterized map) [19,20].

Pedestrian crash-risk locations come as two main types: Intersections and non-intersections [14]. An intersection is defined as the general area where two or more roadways meet [21]. Intersections are locations where most directional changes in travel take place, and consequently where conflicts between pedestrians and vehicles are high [9,22]. While intersection-specific engineering safety measures are used to mitigate these conflicts, intersection design standards often prioritize the operation of vehicles rather than the safety of walkers [8,23]. A non-intersection is any location within a roadway segment or along a transportation facility, that is not at an intersection. Past research has shown that factors (e.g., vehicle type, roadway curves) that impact collisions at intersections do not necessarily impact crashes at non-intersections [14]. 

The identification of crash-risk locations at an intersection and non-intersection area is an important part of modeling pedestrian crashes. However, there are limited definitions, data or methods to appropriately identify crash-risk locations [24]. Regarding intersections, the definition of what constitutes an intersection might be similar in the literature [9,22,25,26,27]. However, the data and methods used to identify intersections vary across studies. For example, while some studies extracted intersection point data from nodes on intersecting roadway polylines [7], other studies conducted field investigations to obtain intersection locations [26,28]. Furthermore, non-intersections have broader definitions that range from highway sections [15,29] to mid-block, cul-de-sac, curve [6], and even toll plazas [13]. 

There are also issues associated with the spatial analysis tools used for measuring features around crash-risk locations [30]. Complex spatial analysis using Geographic Information System (GIS) and advanced quantitative methods are often needed to measure the outcomes (e.g., the number of pedestrian crashes) and predictors (e.g., residential density) of pedestrian crash models [6,7]. Buffering techniques are widely used given the many transportation facilities that include GIS vectors [10]. A wide range of bandwidths have been used for buffering, but most are within a 100 m radius [6,9,31]. 

Regardless of buffer size, overlapping areas between buffers of different crash-risk locations are a major cause of spatial autocorrelation, which impacts the interpretation of pedestrian crash models [6,25,27]. The statistical models estimated in previous studies have been based on the assumption that observations were mutually independent [32,33,34]. However, the statistical requirement that observations be independent and identically distributed (*i.i.d*) is often violated because of the overlapping buffers. Adjacent crash-risk locations are more likely to violate this assumption because they are more likely to have overlapping buffer areas.

Very few studies have accounted for the spatial autocorrelation in pedestrian safety studies. Mixed-effects models have been adopted in some studies to reflect contextual characteristics [6,25,27]. Although these models mitigate the effects of spatial autocorrelation by adopting advanced statistical methods, the source data for spatial analysis units might still involve a problem of autocorrelation derived from overlapping buffers. A different approach is the use of sampling to identify uncorrelated crash-risk locations. For instance, a subset of intersections that were considered to be independent through field investigation can be used in statistical analyses [26,28]. However, this requires extensive time and effort, and typically yields a small sample size.

The objective of this study was to introduce a systematic and replicable protocol to create uncorrelated spatial units of analysis for pedestrian crash modeling for intersection and non-intersection areas. Although the modeling results from previous studies provide valuable insights, measurements of pedestrian crash-risk locations are often not consistent among research projects, in part due to differences in collecting and processing the source data [28]. This has led to complications in interpreting and comparing model results. A standardized method to identify pedestrian crash-risk locations would help improve the reliability, accuracy, and validity of locational factors impacting crash risk. With a clear and replicable unit of analysis for pedestrian crash modeling, researchers and transportation planners could better understand the factors that influence the pedestrian crashes.

## 2. Materials and Methods

### 2.1. Data

#### 2.1.1. Pedestrian Crashes

Pedestrian crash data came from the Transportation Data, GIS and Modeling Office of WSDOT (Washington State Department of Transportation) and covered the years between 2013 and 2017. The data included all crashes that had been reported to and recorded by local police or State Highway Patrol. There were 2222 pedestrian crashes on state routes during the study period, with data including individual-level information such as time, weather, road condition, and socio-demographic and behavioral characteristics of both drivers and pedestrians. In the data, crash location came as milepost on state routes and county roads; and as distance from the closest intersection on city streets. Crash latitude and longitude were identified by WSDOT using Linear Referencing System (LRS) and geocoding tools in GIS.

The data included information as to whether the pedestrian crashes occurred at intersections or non-intersections. The crash data was segmented into nine location types:

- Type 1: At driveway within major intersection;
- Type 2: At intersection and not related;
- Type 3: At intersection and related;
- Type 4: Circulating roundabout;
- Type 5: Exiting roundabout;
- Type 6: At driveway;
- Type 7: Driveway related but not at driveway;
- Type 8: Intersection related but not at intersection;
- Type 9: Not at intersection and not related.

For the forthcoming analysis, this information was re-categorized into 2 groups; intersection (type 1 to 5), and non-intersection (type 6 to 9). Among 2222 state route pedestrian crashes, 1423 (64%) occurred at an intersection and 799 (36%) occurred at a non-intersection. This information was used as a reference to test the performance of the algorithm for detecting unique intersection and non-intersection locations.

#### 2.1.2. Roadway Lanes and Legal Speed Limits

Two transportation network datasets were obtained from the Office of Information Technology of the WSDOT: 1) Roadway lane polyline data, 2) legal speed limit polyline data. First, roadway lane data included state routes, county roads, and city streets. The data included roadway width, number of increasing/decreasing lanes, and milepost information for state routes. Jurisdictional information such as city names were also included for county roads and city streets. There were 18,999 state route segments and 127,652 non-state route roadway segments used in this study. Second, legal roadway speed limit information was obtained as a separate dataset, which contained 2478 records. The data included speed limit information for each state route polyline segment.

#### 2.1.3. Investigating Intersection Point Data

Roadway intersection point data came from the Office of Information Technology of the Washington State Department of Transportation (WSDOT). In this dataset, intersections related to vehicular travel, and were derived not from road or street center lines, but from vehicular traffic lanes. Intersections were defined as any location where vehicular traffic could change travel direction. Different intersections were generated based on traffic direction (e.g., a left-turn lane from the north of an intersection had a different intersection with straight travel lanes than a left turn lane from the south of the same intersection) (NOTE: This definition is generated by the researchers based on their investigation of the data, and has not been confirmed by WSDOT—there is no meta data attached to the intersection dataset). 

The data contained 26,204 records of intersections and provided intersection type information. Nine types were identified:

- Type A: A lane becoming an on ramp to a limited access road;
- Type E: An off-ramp lane to a limited access road;
- Type G: An intersection where roadways are crossed at a common grade;
- Type N: An entrance lane to a limited access road;
- Type O: On and off ramp lanes to limited access roads.
- Type R: Roundabouts;
- Type T: An entrance or an exit lane to a limited access road;
- Type X: An exit lane from a limited access road;
- Type Y: A WYE (Y) connection where lanes formed three legs in the general form of a Y and the angle between 2 legs is less than 60 degrees.

**Figure 1:**
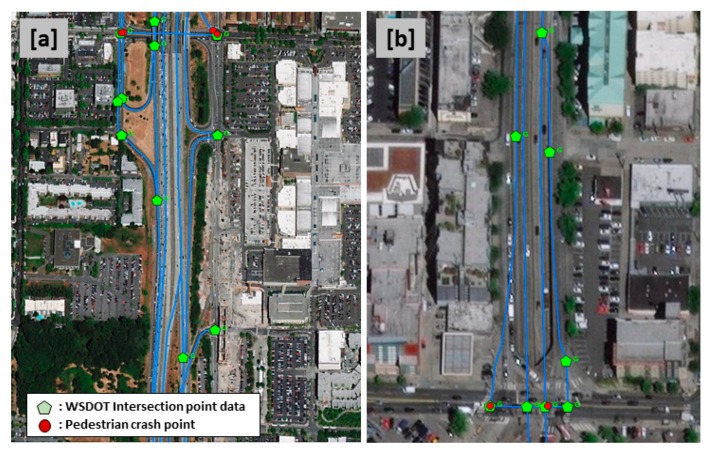
Examples of Washington State Department of Transportation (WSDOT) data identified intersection points in Seattle, at (**a**) (Interstate-5 and NE Northgate Way) and (**b**) (state route 99 and Denny Way) in Seattle, Washington State. The blue lines represent the vehicular lanes in the respective facilities.

Many of the intersection types included in the data did not correspond to locations where pedestrians would cross streets or roads (Figure 1). Two trained GIS analysts investigated over 100 intersection data points and compared them with aerial photos and Google Maps to extract intersection data points where pedestrians could actually walk and cross a street or a road. Overall, they found that only intersection types G (grade intersection) and T (entrance and exit) corresponded to intersections that pedestrian would use. These intersection types were included in the pedestrian intersection data. 

Several observations emerged for intersection type G, and T. Figure 1 shows intersection points identified in the data and pedestrian crash locations. In Figure 1a, Interstate-5 is a limited access highway which pedestrians are prohibited from using. Yet, the facility intersects with locations where pedestrians are allowed to cross. In Figure 1b, lanes from State Route 99, a limited access highway where pedestrians are prohibited to use, intersect with Denny Way, a city street that pedestrians can cross. The data identified 5 points where vehicular lanes intersect. Yet these intersection points are so close to each other that they are representing one pedestrian crossing location.

There were 2 issues identified from these aerial photos. First, in Figure 1a, the WSDOT intersection point data convey information on possible directional changes for vehicles that are not often on or near intersections used by pedestrians. In other words, the intersection point data are not restricted to streets or roads that can be crossed by pedestrians. Thus, the raw intersection GIS data might not be appropriate for modeling pedestrian crashes because some areas are not actually accessible by pedestrians. To model pedestrian crashes, intersection points on limited-access highways (e.g., interstate) needed to be removed before analysis [28]. However, some of these limited-access highways are located near local streets (e.g., city street), where pedestrians are allowed. Hence, a systematic protocol was needed to distinguish the pedestrian accessible intersection points from the inaccessible ones. 

Second, as observed in Figure 1b, when multiple intersection points were in close proximity, intersection locations could be double-counted, leading to double-counting of pedestrian crashes. If crashes were allocated to only one intersection buffer, there would be cases and controls that had similar locational attributes. Furthermore, intersections along a certain corridor will share similar roadway characteristics and land use. In addition, adjacent intersections share similar traffic conditions, and therefore drivers’ behaviors in those locations might also be alike. Thus, crash-risk locations in close spatial proximity were most likely correlated, leading to a biased model [22,27].

### 2.2. Decision Tree Algorithms

**Figure 2:**
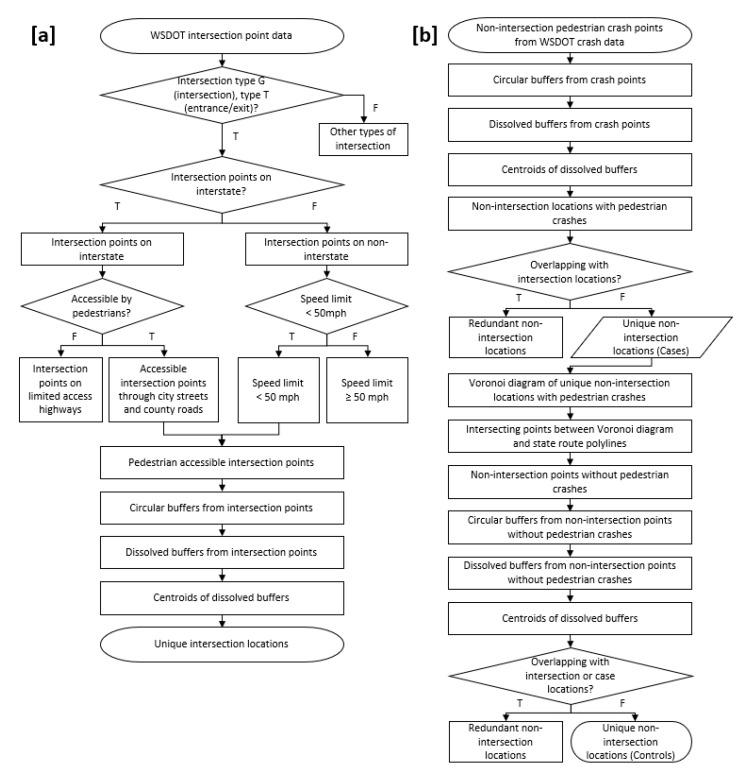
The decision tree algorithms show processes for detecting unique intersection locations (**a**) and non-intersection locations (**b**).

Two algorithms were developed and tested to detect unique crash-risk locations. Figure 2 shows the steps used in the data reduction process. First, an algorithm was created to identify unique intersection locations. Figure 2a is workflow of the algorithm. Intersection point data from WSDOT were used as the input dataset for this process. Pedestrian accessible intersection points were extracted by using intersection type, road type, and legal roadway speed limit information. Figure 2b shows the decisions made for detecting unique non-intersection locations. WSDOT pedestrian crash data were used as a baseline dataset to first identify non-intersection locations with crashes. These locations were considered as the “cases” of a case-control conceptual model. “Control” non-intersection locations were identified using Voronoi diagram techniques to detect random non-intersection locations where crashes did not but could occur. A detailed description for each process is explained in the following sections.

#### 2.2.1. Intersection Points on Limited Access Roadways

From a legal perspective, intersection points on the main thoroughfares of limited–access roadways are not to be used by walkers for transportation purposes. However, in some cases, these intersection points were physically accessible by walkers at ramps and other locations where the highway connected to local streets such as county roads and city streets. In addition, pedestrian crashes have occurred at some of these locations. To identify these locations, 10 m buffers from local streets were created and pedestrian accessible intersection points were extracted.

For the next step, intersection points on non-interstate routes were examined using a state route network dataset, which included legal speed limit information for each roadway segment. State route segments where the speed limits were greater than or equal to 50 mph were used to represent locations inaccessible to pedestrians. Intersections that were beyond 10 m from these segments were identified as being pedestrian-accessible. Pedestrian accessible intersection points on interstate and non-interstate route segments were then merged as a single GIS point layer for detecting unique intersection locations.

#### 2.2.2. Detecting Unique Intersection Locations

**Figure 3:**
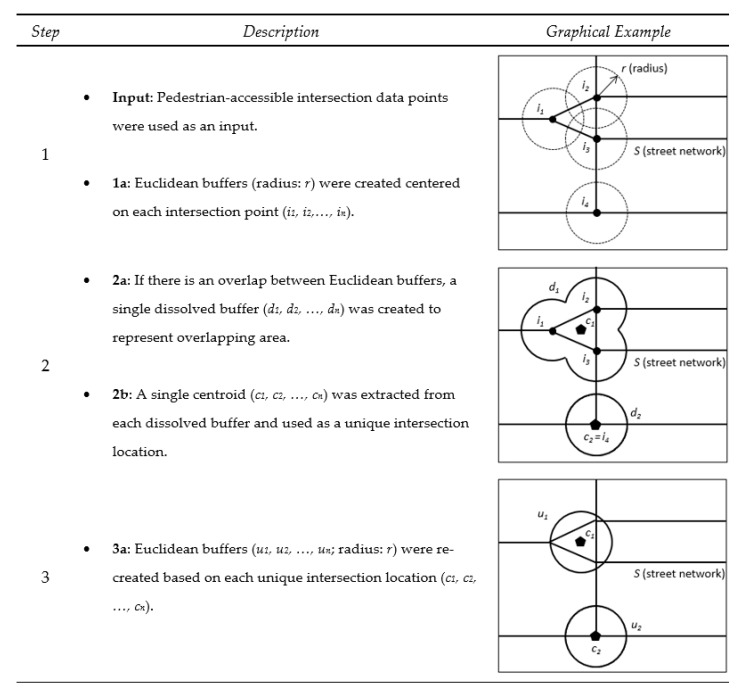
Processing intersection point data and street network for detecting unique intersection locations. Round dot indicates intersection location from the WSDOT state route dataset. Pentagon indicates centroid of unique intersection location.

The creation of Euclidean buffers using state route intersections resulted in many overlapping buffers, leading to potential autocorrelation. Considering 2 intersections with overlapping buffers (i and j), the environmental characteristics of intersection i will be associated with the outcomes (e.g., number of crashes) of intersection j through the overlapping area between the 2 intersection buffers. We applied buffering techniques described in Figure 3 to account for potential autocorrelation. Three steps were used to identify uncorrelated intersection locations without overlaps.

Pedestrian-accessible intersection points identified from the analyses of street network data summarized in Figure 2a were used as an input dataset. Euclidean buffers were first developed from each intersection point to represent initial intersection locations. If there was an overlapping area between Euclidean buffers, a dissolved buffer was created to capture overlapping areas of polygons. A single centroid was then detected from each dissolved buffer and used as a unique intersection location. Lastly, Euclidean buffers were re-created from each point to represent unique intersection locations.

#### 2.2.3. Detecting Unique Non-Intersection Locations

**Figure 4:**
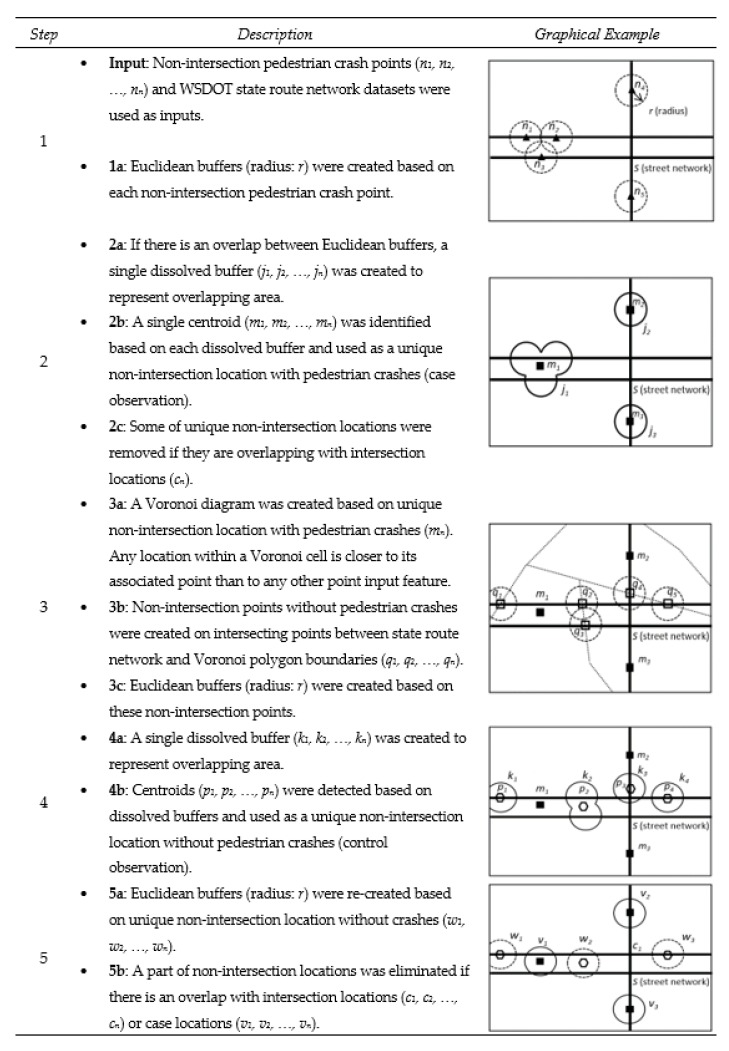
Processing pedestrian crash data and street network for detecting unique non-intersection locations. Black triangle indicates crash locations. Black square indicates non-intersection crash locations (cases); multiple adjacent crash locations are dissolved into one location. Square outline indicates intersection of Voronoi lines with state route and streets. Hexagon outline indicates unique non-intersection locations without crash (controls).

According to the WSDOT pedestrian crash data, 36% of the pedestrian crashes on state routes occurred at non-intersections. The second algorithm was to detect non-intersection locations with (cases) or without (controls) pedestrian crashes. Figure 4 shows the 5 steps used in the analysis. Non-intersection pedestrian crash points on state route network data were first buffered to identify unique non-intersection locations (case observation). Non-intersection locations without pedestrian crashes (control observation) were generated using a Voronoi diagram based on case observation points. The control locations points were positioned where Voronoi polygons boundaries intersected with street segments. Since all Voronoi polygon boundaries represented the farthest lines from the location of cases, the chance of overlaps between cases and controls was minimized. Lastly, to extract unique non-intersection locations removing the overlapping area, the same buffering techniques used in previous steps were repeated.

### 2.3. Parameter Setting and Assessment

The algorithm relies on Euclidean buffers with a defined search radius to identify unique intersection and non-intersection locations. First, the effect of the buffer size on the number of unique crash-risk locations was investigated by looking at the number of locations identified by the algorithm that have and do not have crashes. Second, the overall performance of the algorithm was tested by calculating the proportion of the crashes that were captured by the algorithm-detected crash-risk locations. Lastly, 5 performance runs were generated to test the agreement rates between the algorithm and the locational information from crash data using search radii of 20, 40, 60, 80, and 100 m.

## 3. Results

### 3.1. Effect of Buffer Size on the Number of Unique Crash-Risk Locations

Table 1 shows the number of unique intersection and non-intersection locations based on each search radius setting. The total number of unique intersection locations decreased and the relative proportion of case locations increased as a search radius increased. For example, using a 20 m radius produced 7522 unique intersections with 10.6% having crashes, whereas a 100 m radius produced 3019 unique intersections with 14.2% having crashes. Results of the algorithm for non-intersection data were similar to results with intersection data. The total number of unique non-intersection locations decreased from 1608 at 20 m to 955 at 100 m. The proportion of case (non-intersection location with state route pedestrian crashes) increased from 35.3% at 20 m to 38.7% at 100 m.

### 3.2. Overall Performance of the Algorithms

There were 2222 state route pedestrian crashes in Washington State (2013–2017). Not all of these crashes occurred on algorithm detected intersections and non-intersections. Table 2 shows the number of pedestrian crashes at crash-risk locations and other locations. The proportion of pedestrian crashes occurring at locations not identified using the algorithm was lowest at 20 m (12.8% of crashes) and highest at 100 m (39.2% of crashes). The proportion of pedestrian crashes captured within intersection location buffers ranged between 35.7% (r: 100 m) and 61.4% (r: 40 m). The proportion slightly increased from 20 m to 40 m, then decreased sharply at and above 60 m. The proportion of pedestrian crashes detected by non-intersection location buffers was relatively stable compared to intersection locations. It was lowest at 40 m (22.8% of crashes) and highest at 20 m (27.6% of crashes).

### 3.3. Agreement between Algorithm Crash-risk Locations and Location Type in WSDOT Crash Data

Agreement rates were computed and compared between algorithm-identified locations and WSDOT-recorded crash location. Table 3 shows counts of pedestrian crashes within algorithm-identified intersection location buffers. A total of 1324 pedestrian crashes occurred within 20 m buffer of intersection locations, of which 94.1% identified as intersection-related crashes from the crash data. The agreement rates decreased gradually with increasing buffer radius. The lowest agreement rate (79.2%) was found with 100 m intersection location buffers.

Table 4 shows concurrence between algorithm-identified non-intersection locations and WSDOT data for pedestrian crashes that were recorded as having occurred at non-intersection locations. Of the total of 614 pedestrian crashes within 20 m of non-intersection locations, 98.0% were categorized as non-intersection crashes from the crash data records. The agreement rates showed a sharp decrease after and beyond 60 m. The lowest agreement rate (77.8%) was found with the longest search radius (100 m).

## 4. Discussion

This study produced a systematic and reproducible protocol to identify unique pedestrian-motor-vehicle crash-risk locations at intersection and non-intersection areas. A unit of spatial analysis for pedestrian crash modeling was derived from two algorithms, and the reliability of the protocol was assessed by comparing the outcomes with the actual pedestrian crash data. A set of parameters (buffer sizes) was tested to check the sensitivity of the algorithm results.

Buffer size has a significant impact on the number of unique intersection locations identified using state route intersection points and street network GIS data. The total number of unique intersection locations decreased as the search radius increased because larger buffers create larger overlapping areas around the original intersection points and these areas are then dissolved to identify one crash-risk location. The shortest search radius (20 m) generated 7522 unique intersection locations, down from 26,204 initial intersection points, which meant that on average 3.5 initial intersection points were dissolved as one pedestrian crash-risk location. Although the total number of unique intersection locations was largest with a 20 m buffer radius, the number of actual crash locations identified was largest with a 40 m search radius. Considering that pedestrian crashes are rare events, the determination of a search radius size should be based on the purpose of the study and not only on the number of past crash locations.

Non-intersections are more difficult to identify than intersections because they can be located anywhere along single road segments. By using pedestrian crash data points at non-intersections to construct the Voronoi diagrams, we could detect numerous unique non-intersection locations where a crash could occur. Since any location within a Voronoi polygon is closer to its associated central point than to any other point input feature, newly detected control observations have the farthest distance from case observations, thus minimizing the possibility of overlapping cases and controls. The number of unique non-intersection locations showed a sharp decrease between the 20 m and 40 m search radius, implying that radii shorter than 40 m might be preferable.

The performance of protocol was assessed with over 2,000 pedestrian crashes that occurred on state routes between 2013–2017. Overall, the proportion of pedestrian crashes captured within algorithm-detected locations was highest (87.2% of 2,222 crashes) using the 20 m search radius. Intersection locations captured the largest number of pedestrian crashes with 40 m search radius (61.4%), showing a major decrease after applying a 60 m search radius (47.8%). Non-intersection locations captured relatively steady number of pedestrian crashes (22.8–27.6%).

Agreement rates between algorithm-generated and crash records locations were high, ranging from 79.2% to 94.1% for intersections and 77.8% to 98% for non-intersections, suggesting that the two algorithms could readily distinguish between the two types of locations. Misclassifications were expectedly higher with large buffers, as for instance, would be the case of a non-intersection pedestrian crash occurring close to the intersection location. Clearly, however, pedestrian crash data are a useful reference to assess the protocol, but they are not a gold standard for evaluating the algorithms (e.g., there can be human errors in recording and reporting pedestrian crash locations, and not all crashes are necessarily recorded in the data). 

Given their high performance in identifying two types of crash-risk locations, the algorithms presented in this study have major benefits. First, they can reduce human errors and labor hours to clean existing intersection data similar to the 26,204 intersection points on state routes in Washington State. Second, the algorithm uses clear definitions of and processes to define crash-risk locations. There has been little consistency to-date in identifying non-intersection locations in the literature, which has limited comparisons between studies. Third, the protocol presented come in separate modules that can be applied to data sets that are different from the four publicly accessible datasets used in this study. For instance, some states may not offer intersection point data, in which case researchers will rely on roadway network GIS data to create nodes as intersection points. The buffering techniques modules introduced in this study can be applied to any point data set and help identify unique intersection locations without overlaps. Similarly, modules for creating Voronoi polygons can be used to detect non-intersection locations on any point data set. Lastly, while only one Voronoi diagram was used to identify non-intersection locations in this study, this module can be repeated multiple times to create more control observations. Depending on the purpose of a study or the data availability, the algorithms offer a useful way to create a balanced sample of crash-risk locations.

The study has limitations. First, the reduction of intersection points performed by the algorithms was evaluated with multiple criteria (e.g., sample size, coverage, and accuracy), but additional manual check for randomly selected data points (e.g., comparison with aerial photos) might enhance the protocol. Second, the algorithm for non-intersection locations relies on empirical pedestrian crash data, which may not be available everywhere or which may be of poor quality. Consistency of the pedestrian crash data across jurisdictions will make this algorithm more useful.

## 5. Conclusions

The protocol developed in this study provides an efficient and effective way to create spatial units of analysis for pedestrian crash modeling. It can save substantial time in identifying unique intersection and non-intersection locations. The algorithms will also make it possible for researchers to compare their model results with other studies by using the same unit of analysis.

The algorithms showed sustained performance in identifying crash-risk locations at road or street intersections and non-intersections. Different search radii serve to optimize sample size, coverage, and accuracy, depending on the objective of a study. In the present analyses, the algorithms using the 20 m buffer showed highest performance, and a sharp drop in performance was noted when using 60 m and larger buffers. A 40 m radius can be an alternative if large numbers intersection locations are desired. Buffers between 20 and 40 m should be tested in future studies.

The protocol discussed in this study is a tool for integrating pedestrian crash data with the transportation network and for detecting unique intersection and non-intersection locations. Pedestrian crash modeling using this protocol will broaden the applicability of algorithms and enrich the discussion in the future.

## Figures and Tables

**Table 1:** Count of unique intersection locations by buffer size.

Parameter	20 m	40 m	60 m	80 m	100 m
Unique Intersection					
With recorded crash	794 (10.6%)	818 (12.3%)	596 (12.8%)	514 (13.8%)	428 (14.2%)
With no recorded crash	6728 (89.4%)	5808 (87.7%)	4056 (87.2%)	3212 (86.2%)	2591 (85.8%)
Total	7522	6626	4652	3726	3019
Unique Non-intersection					
With recorded crash	567 (35.3%)	455 (38.0%)	419 (38.3%)	374 (37.9%)	370 (38.7%)
With no recorded crash	1041 (64.7%)	743 (62.0%)	674 (61.7%)	612 (62.1%)	585 (61.3%)
Total	1608	1198	1093	986	955

**Table 2:** Number of pedestrian crashes at crash-risk locations and other locations.

Parameter	20 m	40 m	60 m	80 m	100 m
Others (not captured by crash-risk locations)	284 (12.8%)	351 (15.8%)	629 (28.3%)	784 (35.3%)	781 (39.2%)
Within intersection location buffers	1324 (59.6%)	1364 (61.4%)	1063 (47.8%)	928 (41.8%)	793 (35.7%)
Within non-intersection location buffers	614 (27.6%)	507 (22.8%)	530 (23.9%)	510 (23.0%)	558 (25.1%)
Total	2222	2222	2222	2222	2222

**Table 3:** Agreement rates between algorithm intersection location and crash data.

Buffer Radius (*r*)	Count of Pedestrian Crashes			
	All Algorithm Intersection Crash Locations	Location Type from WSDOT Crash Data		Agreement
		Intersection	Non-Intersection	
20 m	1,324	1246	78	94.1%
40 m	1,364	1202	162	88.1%
60 m	1,063	907	156	85.3%
80 m	928	761	167	82.0%
100 m	793	628	165	79.2%

**Table 4:** Agreement rates between non-intersection location and crash data.

Buffer Radius (*r*)	Count of Pedestrian Crashes			
	All Algorithm Non-Intersection Crash Locations	Location Type from WSDOT Crash Data		Agreement
		Intersection	Non-Intersection	
20 m	614	12	602	98.0%
40 m	507	11	496	97.8%
60 m	530	65	465	87.7%
80 m	510	86	424	83.1%
100 m	558	124	434	77.8%

## References

[1] Millward H., Spinney J., Scott D. (2013). Active-transport walking behavior: Destinations, durations, distances. J. Transp. Geogr..

[2] Ogilvie D., Foster C.E., Rothnie H., Cavill N., Hamilton V., Fitzsimons C.F., Mutrie N. (2007). Interventions to promote walking: Systematic review. BMJ.

[3] Tight M., Timms P., Banister D., Bowmaker J., Copas J., Day A., Drinkwater D., Givoni M., Gühnemann A., Lawler M. (2011). Visions for a walking and cycling focussed urban transport system. J. Transp. Geogr..

[4] Pucher J., Dijkstra L. (2003). Promoting Safe Walking and Cycling to Improve Public Health: Lessons from The Netherlands and Germany. Am. J. Public Health.

[5] Pedestrian Traffic Fatalities by State: 2017 Preliminary Data|GHSA. https://www.ghsa.org/resources/spotlight-pedestrians18.

[6] Quistberg D.A., Howard E.J., Ebel B.E., Moudon A.V., Saelens B.E., Hurvitz P.M., Curtin J.E., Rivara F.P. (2015). Multilevel models for evaluating the risk of pedestrian-motor vehicle collisions at intersections and mid-blocks. Accid. Anal. Prev..

[7] Moudon A.V., Lin L., Jiao J., Hurvitz P., Reeves P. (2011). The risk of pedestrian injury and fatality in collisions with motor vehicles, a social ecological study of state routes and city streets in King County, Washington. Accid. Anal. Prev..

[8] Lee C., Abdel-Aty M. (2005). Comprehensive analysis of vehicle–pedestrian crashes at intersections in Florida. Accid. Anal. Prev..

[9] Pulugurtha S.S., Sambhara V.R. (2011). Pedestrian crash estimation models for signalized intersections. Accid. Anal. Prev..

[10] Schneider R.J., Diógenes M.C., Arnold L.S., Attaset V., Griswold J., Ragland D.R. (2010). Association between Roadway Intersection Characteristics and Pedestrian Crash Risk in Alameda County, California. Transp. Res. Rec. J. Transp. Res. Board.

[11] Yu C.-Y. (2015). How Differences in Roadways Affect School Travel Safety. J. Am. Plan. Assoc..

[12] Al-Ghamdi A.S. (2002). Using logistic regression to estimate the influence of accident factors on accident severity. Accid. Anal. Prev..

[13] Abdel-Aty M. (2003). Analysis of driver injury severity levels at multiple locations using ordered probit models. J. Saf. Res..

[14] Moore D.N., Schneider W.H., Savolainen P.T., Farzaneh M. (2011). Mixed logit analysis of bicyclist injury severity resulting from motor vehicle crashes at intersection and non-intersection locations. Accid. Anal. Prev..

[15] Milton J., Mannering F. (1998). The relationship among highway geometrics, traffic-related elements and motor-vehicle accident frequencies. Transportation.

[16] Nie K., Wang Z., Du Q., Ren F., Tian Q., Nie K., Wang Z., Du Q., Ren F., Tian Q. (2015). A Network-Constrained Integrated Method for Detecting Spatial Cluster and Risk Location of Traffic Crash: A Case Study from Wuhan, China. Sustainability.

[17] Huang H., Abdel-Aty M.A., Darwiche A.L. (2010). County-Level Crash Risk Analysis in Florida: Bayesian Spatial Modeling. Transport Res. Rec..

[18] Wier M., Weintraub J., Humphreys E.H., Seto E., Bhatia R. (2009). An area-level model of vehicle-pedestrian injury collisions with implications for land use and transportation planning. Accid. Ana. Prev..

[19] Xie K., Ozbay K., Kurkcu A., Yang H. (2017). Analysis of Traffic Crashes Involving Pedestrians Using Big Data: Investigation of Contributing Factors and Identification of Hotspots. Risk Anal..

[20] Xie Z., Yan J. (2008). Kernel Density Estimation of traffic accidents in a network space. Comput. Environ. Urban Syst..

[21] Hancock M.W., Wright B. (2013). A Policy on Geometric Design of Highways and Streets.

[22] Wang X., Abdel-Aty M., Brady P. (2006). Crash Estimation at Signalized Intersections: Significant Factors and Temporal Effect. Transp. Res. Rec. J. Transp. Res. Board.

[23] Pietrucha M.T., Opiela K.S. (1993). Safe accommodation of pedestrians at intersections. Transport Res. Rec..

[24] Forsyth A., Van Riper D., Larson N., Wall M., Neumark-Sztainer D. (2012). Creating a replicable, valid cross-platform buffering technique: The sausage network buffer for measuring food and physical activity built environments. Int. J. Health Geogr..

[25] Mitra S. (2009). Spatial Autocorrelation and Bayesian Spatial Statistical Method for Analyzing Intersections Prone to Injury Crashes. Transp. Res. Rec. J. Transp. Res. Board.

[26] Poch M., Mannering F. (1996). Negative Binomial Analysis of Intersection-Accident Frequencies. J. Transp. Eng..

[27] Guo F., Wang X., Abdel-Aty M.A. (2010). Modeling signalized intersection safety with corridor-level spatial correlations. Accid. Anal. Prev..

[28] Forsyth A., Schmitz K.H., Oakes M., Zimmerman J., Koepp J. (2006). Standards for Environmental Measurement Using GIS: Toward a Protocol for Protocols. J. Phys. Act. Health.

[29] Shankar V.N., Ulfarsson G.F., Pendyala R.M., Nebergall M.B. (2003). Modeling crashes involving pedestrians and motorized traffic. Saf. Sci..

[30] Satria R., Castro M. (2016). GIS Tools for Analyzing Accidents and Road Design: A Review. Transp. Res. Procedia.

[31] Ma L., Yan X., Qiao W. (2014). A Quasi-Poisson Approach on Modeling Accident Hazard Index for Urban Road Segments. Discret. Dyn. Nat. Soc..

[32] Aguero-Valverde J., Jovanis P.P. (2006). Spatial analysis of fatal and injury crashes in Pennsylvania. Accid. Anal. Prev..

[33] Song J., Ghosh M., Miaou S., Mallick B. (2006). Bayesian multivariate spatial models for roadway traffic crash mapping. J. Multivar. Anal..

[34] Quddus M.A. (2008). Modelling area-wide count outcomes with spatial correlation and heterogeneity: An analysis of London crash data. Accid. Anal. Prev..

